# ‘Standardized patients’ in teaching the communication skill of history-taking to four-year foreign medical undergraduates in the department of obstetrics and gynaecology

**DOI:** 10.1186/s12909-019-1541-y

**Published:** 2019-04-15

**Authors:** Jing Zhang, Meng Cheng, Na Guo, Aiyun Xing, Liangzhi Xu

**Affiliations:** 1 Department of Obstetrics and Gynaecology, West China Second University Hospital, Sichuan University, Chengdu, Sichuan People’s Republic of China; 2 Reproductive Endocrinology and Regulation Laboratory, West China Second University Hospital, Sichuan University, Chengdu, Sichuan People’s Republic of China; 3 Key Laboratory of Birth Defects and Related Diseases of Women and Children, Sichuan University, Ministry of Education, Chengdu, People’s Republic of China

**Keywords:** Standardized patient, History-taking, Communication skill, Foreign students, Medical undergraduate, Obstetrics and gynaecology, Case report

## Abstract

**Background:**

Many foreign students have difficulty taking histories from Chinese patients, especially in clinical context of the Department of Obstetrics and Gynaecology. The efficacy of using standardized patients to prepare foreign students for communicating with Chinese patients and taking their histories was evaluated in this study.

**Methods:**

Ninety-four four-year foreign students were assigned to one of three clinical sub-departments (gynaecology, obstetrics, and reproductive endocrinology) to practice history-taking; after practicing in one sub-department, the students were then crossed over to a different department. The histories were taken from real patients in the sub-departments of obstetrics and reproductive endocrinology and from standardized patients in the sub-department of gynaecology. Prior to contact with real patients in the sub-department of reproductive endocrinology, the students practised with standardized patients. The quality levels of the case reports generated in the three departments were compared by repeated measures ANOVA. The attitudes, satisfaction and suggestions of the students were also investigated through a questionnaire.

**Results:**

The local Chinese language spoken by the patients was thought to be the most common difficulty students (76.7%) encountered while taking patient histories. Two-thirds and one-third of the students were interested in taking histories from standardized and real patients, respectively. Most students (94.2%) thought that working with standardized patients was useful for practising communication skills with Chinese patients. The total scores of the case reports were significantly different among the three groups (*P* < 0.001), and compared with case reports collected from real patients, case reports collected from standardized patients were of better quality. However, the quality of the case reports taken from real patients was better when the case reports were generated by students who had previous practice with standardized patients than when they were generated by students lacking such experience (*P* < 0.001).

**Conclusions:**

Standardized patient training for practising history-taking can be included as part of the clinical training curriculum for foreign medical undergraduates in the Department of Obstetrics and Gynaecology in China.

**Electronic supplementary material:**

The online version of this article (10.1186/s12909-019-1541-y) contains supplementary material, which is available to authorized users.

## Background

Before arriving at a diagnosis, doctors proceed sequentially through the stages of history-taking, physical examination and supplementary testing. History-taking provides the foundation on which diagnosis, management and doctor-patient relationships are based and is a fundamental clinical skill.

Good communication between doctors and patients has positive effects on history-taking and patient management. To “maintain and communicate accurate patient medication information” is cited by the Joint Commission as a national patient safety goal in a variety of healthcare settings [1]. History-taking training is an important part of medical education and continuing medical education in many countries, and an important learning objecting is how to conduct patient interviews to gather patient information. In particular, during their clinical education, clinical medical students should be trained how to handle difficult situations that may arise during patient-doctor encounters. Different educational methods of teaching history-taking skills to medical students have been found to be effective, such as small group workshops that include role playing and interviews with real patients, after which feedback is provided that includes a review of the recorded session. Students in the early preclinical training stage might profit from approaches that help them focus on interview skills, removing the distraction of thinking about differential diagnoses or clinical management [2].

With growing international communication, an increasing number of foreign students are choosing to study clinical medicine in China. In 2001, a representative total class size of foreign students in Chinese medical colleges was only 23. In 2016, the number of foreign students increased to 96 per class. Now, more foreign students are choosing to remain in China for their clinical practice internships rather than returning to their home countries. After they complete their internships and graduate from medical school, they return to their home countries to complete their residencies. Therefore, faculty members have begun receiving feedback from site preceptors that many foreign students have difficulty effectively communicating with local patients during history-taking, especially in the Departments of Obstetrics and Gynaecology.

The clinical symptoms and signs of patients in the Department of Obstetrics and Gynaecology are related to the reproductive organs and the history of childbearing and menstruation and involve problems with the vagina and vulva, including the patient’s history of vaginal delivery and induced abortion; these are subjects that many patients consider private. Most Chinese women feel too awkward to talk about these issues with foreign students. Therefore, foreign students should be offered many more opportunities to practice their communication skills, and innovative methods of teaching history-taking should be employed within a safe learning environment before the students are asked to take patient histories in a real-world setting. The repeated practice of these skills may improve students’ confidence in effectively taking medical histories. As part of students’ medical education, medical schools often use standardized patients (SPs) to depict realistic patient interactions and presentations of disease. By conducting interviews with SPs, medical students learn how to communicate with patients in a situation that does not require the use of actual patients [3].

This article reports the results of a pilot training course that used SPs to train foreign students in the Department of Obstetrics and Gynaecology in the spring semester of 2016. Based on the key teaching objectives of the pilot program, this article focuses on the learning needs of foreign students and their history-taking competency levels after receiving training according to the three different teaching methods. The central issue is whether the students’ competencies are better when they are trained with SPs or real patients. In addition, the key method of using SPs was evaluated by the students who were asked to assess their satisfaction level and impressions.

## Methods

### Setting and participants

The study was conducted at a major urban university teaching hospital located in southwestern China that serves as a tertiary care facility and referral centre for children and women.

Ninety-four four-year foreign students starting their main clinical term of the six-year curriculum were included in the present study; two students were absent from clinical training in the Department of Obstetrics and Gynaecology. The included students had already attended, in their third year, lectures on diagnostics and doctor-patient communication skills. These students had prior clinical exposure and were also afforded the opportunity to take histories in other fields, such as internal medicine. By the spring semester of 2016, these four-year students had completed eight weeks of lectures on theoretical knowledge and a video-assisted course showing how to communicate with patients in the Department of Obstetrics and Gynaecology.

Following this initial training, the students were divided into three groups. Each of the three groups received clinical training and practiced medical history-taking in one of the following clinical sub-departments of the Department of Obstetrics and Gynaecology: gynaecology, obstetrics, and reproductive endocrinology. After practicing in one sub-department, the groups crossed over to the other departments. Each group practised history-taking in all three departments and was guided by three attending doctors in each department.

### Teaching methods

To provide the four-year medical students with adequate opportunities to practice medical history-taking and communication, every student in each group was required to take histories in each department by 1) working directly with real obstetrical patients (real patients group, R), 2) working with the gynaecological standardized patients, who were played by attending doctors according to set clinical scenarios (standardized patients group, SP), and 3) practising first with infertile SPs and then taking histories from real infertile patients (SP + R).

For the SP practice, the authors developed a 2-h group course with SPs who were played by site preceptors with simulated features (portraying patients during interviews with the medical students during history-taking training) in the Department of Obstetrics and Gynaecology. Role playing was adopted in the SP practice, with the site preceptors acting as the patients and the students acting as the doctors. Real clinical scenarios related to infertility and uterine myoma were prepared by faculty members who were not involved in the role playing during the SP practice. The site preceptors first stated the purpose of the exercise and then introduced the method involving SPs. Fifteen or sixteen students participated in the simulated activity each time. One of the students assumed the role of doctor, while a female site preceptor assumed the role of a patient (the SP scenarios were based on real clinical cases). The other students observed the role playing activity and later provided feedback. The facilitators monitored the reactions of the students and answered the students’ questions. Every student was required to write their own case report for the case of uterine myoma portrayed by the SP.

For the real clinical practice, groups of 4 to 5 students were assigned to different clinical wards and were expected to take medical histories from real patients under the supervision of the site preceptor. Every student was required to write their own case report in the sub-departments of obstetrics (R) and reproductive endocrinology (SP + R). The quality levels of the case reports generated under the three different conditions (R, SP + R, SP) were compared to assess the efficacy of the three teaching methods for practising history-taking. Finally, feedback was given to the students about the areas of the assessment in which they did not perform well on and how they could improve.

### Questionnaire survey

Participating students who completed the medical history-taking training were invited to complete an anonymous questionnaire (Additional file 1) that was administered by the faculty who did not participate in the teaching or SP role-playing sessions. The faculty explained the voluntary nature of the survey and provided an estimate of the time that it would take to complete it. The questionnaires were designed to survey the difficulties that the students faced in history-taking as well as their attitudes, satisfaction and suggestions for other teaching methods to use. The students were also queried about their nationality, language, and the duration of their stay in China.

### Quality comparison of case reports

After the courses, three faculty members who were blind to the details of the teaching methods used in each sub-department (R in obstetrics, SP in gynaecology, SP + R in reproductive endocrinology) individually assessed the quality of the case reports written by the students. The evaluation criteria belonged to six categories containing forty-five items. For example, recording the chief complaint, including the main symptom and duration, was assessed on a 4-point scale (absolutely accurate, somewhat accurate, somewhat inaccurate or absolutely inaccurate), with “3” and “0” indicating the highest and lowest levels of accuracy for the content of the case reports, respectively. The scores of the case reports were compared among the three categories of teaching methods to evaluate the efficacy of using SPs to teach communication skills and history-taking.

### Statistical analysis

The data from the questionnaire survey were descriptive and are presented as numbers (n) and percentages (%). Repeated measures ANOVA was performed to determine whether there were significant differences among the mean scores for the three methods of practising history-taking. The data are presented as the means and standard deviations. The results were entered into Microsoft Excel (Microsoft Corp., Richmond, WA, USA) and analysed using SPSS version 17.0 (SPSS 17.0).

## Results

Of the 94 students taking medical histories, only 86 (91.5%) completed the survey. Most of the students were from Southeast Asia, particularly India. The local Chinese language spoken by the patients was the biggest difficulty faced by most foreign students (76.7%) when collecting histories. Nearly one-third of the students (37.2%) thought that ‘cannot speak the Chinese Putong language themselves’ made history-taking difficult. Only a few students (4.7%) thought other factors, such as ‘being unfamiliar with Chinese medical terms’ and ‘patients were not cooperative enough’, were the main difficulties confronted in history-taking. Few students (3.5%) thought ‘being unfamiliar with OB/GY diseases’ was the main difficulty.

Two-thirds and one-third of students were interested in taking histories from SPs and real patients, respectively, but they were not interested in learning history-taking from a video (the video-assisted course showing how to communicate with patients that they had attended in their third year).

Most students (94.2%) thought that working with SPs was useful for practising communication skills with Chinese patients and that the method of practising with SPs particularly aided them in collecting accurate histories. Clinical apprenticeships serve to help students learn, understand and apply the structure and strategy of history-taking while improving their interviewing skills with the patients in the Department of Obstetrics and Gynaecology. After practising with SPs, the students become aware that menstrual and childbearing histories are especially important for female patients in the Department of Obstetrics and Gynaecology. Although some female Chinese patients might be too embarrassed to talk about those histories, it is important for doctors to ask for the relevant details.

Students provided several suggestions for how to improve the quality of clinical teaching in the Department of Obstetrics and Gynaecology. We think that many of them are feasible, except for “preparing real patients who can speak English.” The results of the questionnaire are listed in Table 1.

**Table 1:** The results of questionnaire survey in foreign medical students

Categories	Items	NO.	Percentage
Countries	India	64	74.4%
	Srilanka	11	12.8%
	Malaysia	6	7.0%
	Thailand	3	3.5%
	Canada	1	1.2%
	Indonesia	1	1.2%
Difficulties for students in taking history	Can’t understand local language	66	76.7%
	Can’t speak Chinese Putong language	32	37.2%
	Being unfamiliar with the diseases of OB/GY	3	3.5%
	Others	4	4.7%
Interested teaching method	Taking history from standardized patients	55	64.0%
	Taking history from real patients	27	31.4%
	Practicing Chinese with teachers on courses	9	10.5%
	Watching videos on taking history in OB/GY	3	3.5%
The efficacy of standardized patient on communication skill	Very useful	45	52.3%
	Partly useful	36	41.9%
	Useless	5	5.8%
Aspects that the standardized patient can help in	Training skill in collecting history	75	87.2%
	Helping arranging the supplementary tests	23	26.7%
	Helping diagnosing the diseases	29	33.7%
	Helping treating the disease	7	8.1%
In which language the students want the teachers speak	English	55	64.0%
	Both English and Chinese	33	38.4%
	Chinese	0	0.0%
Suggestion for improving the clinical teaching methods	More practice in taking history both from SP and real patients	19	22.1%
	Get more help from teachers or others who are familiar with the Chinese or local language	12	14.0%
	Prepare real clinical patients who can speak English	10	11.6%
	Discuss the clinical cases again to make sure the students have collected the right information	8	9.3%
	Provide booklet containing the outline of history taking in Chinese, Pinyin and English	7	8.1%

In total, 282 case reports in three sub-departments (obstetrics, gynaecology, and reproductive endocrinology) were generated by 94 students. The quality scores of the case reports assigned by the three faculty members were compared, and the results are listed in Table 2. In general, the total scores of the case reports and the scores for chief complaint, present history, past history, personal history, menstrual and childbearing history and family history were all significantly different among the three teaching methods (*P* < 0.001).

**Table 2:** The comparison for scores of case reports among three teaching methods

Indexes	Methods^a^	Mean	SD	95%CI		Mean square	F	P
				Lower	Upper			
Total score	Real patients	101.28	1.12	99.06	103.49	6644.44	86.78	< 0.001*
	SP + real patients	108.32	1.05	106.23	110.40			
	SP^b^	118.02	0.74	116.55	119.49			
Chief complain	Real patients	5.50	0.09	5.31	5.69	89.84	136.14	< 0.001^*^
	SP + real patients	5.48	0.08	5.32	5.64			
	SP	4.00	0.00	4.00	4.00			
Present history	Real patients	26.04	0.43	25.20	26.89	985.38	95.83	< 0.001^*^
	SP + real patients	31.85	0.37	31.12	32.59			
	SP	26.47	0.20	26.07	26.87			
Past history	Real patients	14.60	0.26	14.08	15.11	161.17	33.57	< 0.001^*^
	SP + real patients	15.99	0.22	15.56	16.42			
	SP	17.21	0.21	16.79	17.63			
Personal history	Real patients	12.98	0.22	12.54	13.42	1117.03	191.25	< 0.001^*^
	SP + real patients	11.77	0.33	11.12	12.42			
	SP	17.69	0.14	17.42	17.96			
Menstrual and childbearing	Real patients	33.37	0.53	32.32	34.43	2215.11	151.49	< 0.001^*^
	SP + real patients	31.88	0.36	31.17	32.59			
	SP	40.94	0.37	40.21	41.66			
Family history	Real patients	8.79	0.18	8.43	9.14	239.112	99.48	< 0.001^*^
	SP + real patients	11.35	0.19	10.97	11.74			
	SP	11.71	0.12	11.48	11.95			

^*^P<0.05, Repeat measures ANOVA

^a^Ninety four case reports were collected in each group respectively

^b^SP, standardized patients

**Fig. 1:**
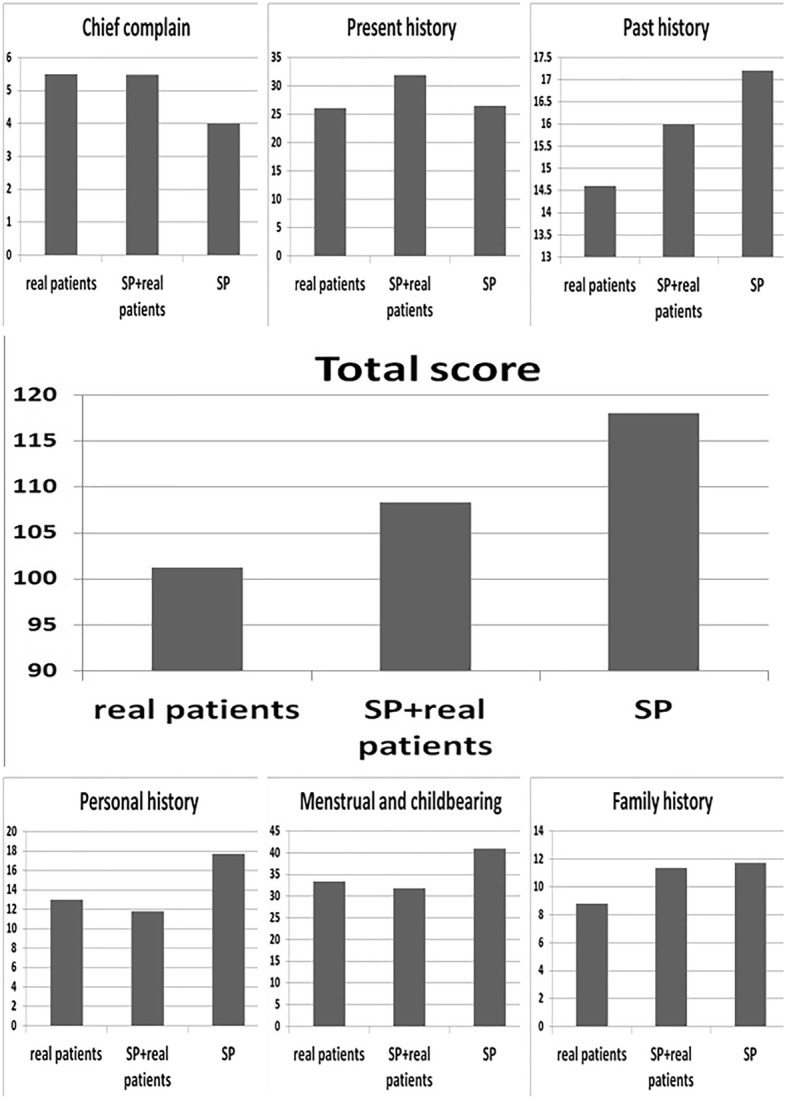
The scores of case reports for the three different history-taking training methods

The quality scores of the case reports generated by students working with only SPs were better than those of the reports generated by students working with real patients (Table 3 and Fig. 1). However, the quality scores of the case reports taken from real patients by student with previous experience working with SPs was better than the quality scores of the reports taken by students who lacked that experience (*P* < 0.001), which suggests that SP + R is a more effective teaching method than R. First practising with SPs led to significantly better scores regarding the collection of the present history (*P* < 0.001), past history (*P* < 0.001) and family history (*P* < 0.001) information; no difference in scores regarding the collection of information about the chief complain (*P* = 0.87); and much worse scores regarding collecting information about the personal history (*P* = 0.001) and menstrual and childbearing history (*P* = 0.009). The quality scores of case reports generated from histories taken from only SPs were much better than those of the reports generated from histories taken from real patients (R, SP + R), especially with regard to the past, personal, menstrual and childbearing histories.

**Table 3:** Pairwise comparison for the quality of case reports of any two teaching methods

Indexes	Method A^a^	Method B	Mean differences (A-B)	SE	P	95%CI of mean differences	
						Lower	Upper
Total score	Real patients	SP + real patients	−7.04	1.19	< 0.001*	−9.42	−4.66
	Real patients	SP	−16.75	1.27	< 0.001^*^	− 19.27	− 14.22
	SP^b^	SP + real patients	9.70	1.35	< 0.001^*^	7.01	12.39
Chief complain	Real patients	SP + real patients	0.02	0.13	0.87	−0.24	0.28
	Real patients	SP	1.50	0.09	< 0.001^*^	1.31	1.69
	SP	SP + real patients	−1.48	0.08	< 0.001^*^	−1.64	−1.32
Present history	Real patients	SP + real patients	−5.81	0.51	< 0.001^*^	−6.81	−4.81
	Real patients	SP	−0.43	0.48	0.381	−1.39	0.53
	SP	SP + real patients	−5.38	0.41	< 0.001^*^	−6.19	−4.57
Past history	Real patients	SP + real patients	−1.39	0.35	< 0.001^*^	−2.08	−0.71
	Real patients	SP	−2.62	0.29	< 0.001^*^	−3.20	− 2.03
	SP	SP + real patients	1.22	0.32	< 0.001^*^	0.59	1.85
Personal history	Real patients	SP + real patients	1.21	0.36	0.001^*^	0.50	1.92
	Real patients	SP	−4.71	0.23	< 0.001^*^	−5.17	− 4.26
	SP	SP + real patients	5.93	0.36	< 0.001^*^	5.22	6.63
Menstrual and childbearing	Real patients	SP + real patients	1.49	0.56	0.009^*^	0.38	2.60
	Real patients	SP	−7.56	0.59	< 0.001^*^	−8.75	−6.38
	SP	SP + real patients	9.05	0.51	< 0.001^*^	8.04	10.07
Family history	Real patients	SP + real patients	−2.56	0.24	< 0.001^*^	−3.03	−2.10
	Real patients	SP	− 2.923	0.21	< 0.001^*^	−3.34	−2.51
	SP	SP + real patients	0.36	0.23	0.124	−0.10	0.83

^*^P<0.05, Repeat measures ANOVA

^a^Ninety four case reports were collected in each group respectively

^b^SP, standardized patients

## Discussion

The study indicates that the local Chinese language is the most common difficulty facing foreign students when collecting histories. SP training is perceived by the students to be an effective method of teaching history-taking to foreign medical students before coming into contact with real Chinese patients in the Department of Obstetrics and Gynaecology; this perception was validated in this study.

The results of the survey demonstrated that students face unique challenges in doctor-patient communication because they do not have sufficient clinical experience. Furthermore, for foreign students, the challenges are much greater because they may be unfamiliar with the local language, culture or customs in China. The following factors are also recognized as barriers to history-taking faced by our foreign medical students: 1) patients may provide misleading face-to-face reports to foreign students because of fear or embarrassment; 2) patients can be inconsistent in their recollection of events, which is caused by difficulties in communication, comprehension, recall and evaluation [4, 5]; and 3) because of the language barrier and cultural differences, foreign students use medical jargon that can confuse patients, which leads to inappropriate variations in questions and constitutes a barrier to collecting a more salient medical history.

Bradley et al. argued that difficulties in the effective delivery of healthcare most often result from problems in communication between patients and doctors rather than from any failing in the technical aspects of medical care [6]. Medical students are faced with unique challenges in doctor-patient communication because they do not have pre-existing relationships with their patients. Students do not always encounter patients who are respectful, polite or timid. One of the purposes of a clinical apprenticeship is to help students learn, understand and apply the structure and strategy of history-taking and to use it to improve their interviewing skills throughout their medical careers. Previously, medical education failed to place sufficient emphasis on doctor-patient communication, according more importance to the disease than to the patient. Unfortunately, not enough time is allocated for students to talk to, listen to and understand patients, even though such activities represent a fundamental aspect of any treatment [7].

It is clear that medical schools must implement in their teaching curriculum a much stronger method for helping and motivating foreign undergraduates with regard to history-taking. For example, providing a booklet listing key points of history-taking for typical diseases that is annotated in Chinese, Pinyin and English is a useful method. Focused, intense training is another helpful method. In the future, we can provide more opportunities for foreign students to practice history-taking. In light of the concerns raised about language barriers faced by students, we can arrange Chinese students who speak both English and Chinese fluently to help with collecting histories.

Currently, students are not very interested in what they view as boring and instructor-centred educational methods [8]. Therefore, there is an increasing emphasis on student-centred education and role-playing methods, such as the one employed in the present study. Most of the students (94.2%) participating in the present study thought that SPs were useful for practising communication skills with Chinese patients, particularly with regard to accurate history-taking. Two-thirds and one-third of the students were interested in taking histories from SPs and real patients, respectively, but they were not interested in learning methods of taking histories from a video. It seems that students prefer to participate directly in clinical practice.

The use of SPs, who constitute an educational tool with simulated features, builds knowledge and skills when it is used properly and is enjoyable for the participants. The simulated environment allows healthcare educators to create real-life scenarios that facilitate student learning while decreasing student stress because they do not have real-life consequences. We think that this also explains our finding that the quality of case reports collected from SPs is better than that of reports collected from real patients. The SPs were played by attending doctors in the present study. All the attending doctors had interviewed the SPs to practise history-taking and physical examinations when they were college students. Therefore, they were familiar with both the role of the SPs and the clinical characteristics of patients in the Department of Obstetrics and Gynaecology. The ‘patients’ simulated by them were much more emotional, representing the severe anxiety experienced by patients with infertility, which might be difficult for real patients to express. However, SPs simulated by attending doctors might be much easier for foreign students to understand because they might be speaking more understandably as patients. We think this might be another reason for the better quality of the case reports collected from SPs compared with those collected from real patients. The use of SPs in medical education is becoming more prevalent as instructors look to create innovative ways of delivering course material. The positive evaluation of SPs as a didactic and methodical practice has been reported previously [9–11]. This finding is consistent with the results of the present study that showed that practising SP first improved the quality of case reports taken from real patients. However, additional exposure to the clinical cases in the group interviewing standardized patients + real patients (SP + R) might also be the reason for the better quality of the case reports when compared to those generated by the students interviewing only real patients. This confounding factor could be resolved by comparing the methods of SP + R and R + R in the future.

Teaching communication skills and assessing whether these skills have been taught successfully are not easy tasks. Furthermore, considering the cultural characteristics of the patient will allow patient-doctor communication to be performed in a more mutual and cooperative way [12]. The cultural diversity of present-day China necessitates doctors who are capable of communicating in a culturally sensitive manner. In the future, education programmes and courses for students should also include information on what they should do and how they should behave as doctors when they are faced with difficult, rude, disrespectful, or forceful patients.

The purpose of the present study was to evaluate the efficacy of using SPs to improve communication skills and to compare the quality of case reports generated after training with different teaching methods. Therefore, only after teachers had evaluated the case reports did the students receive feedback from their teachers on the quality of their reports.

One limitation of this study was the method used to assess the efficacy of the case report training, especially in light of the concerns regarding language barriers raised by the students. Although the teachers assessing the case reports had been trained in advance, subjective assessment bias could still exist. In the future, an objective, structured clinical examination to evaluate the quality of case reports can be explored to resolve this issue. In addition, in the future, the scores can be evaluated by both SPs and the foreign students to assess the efficacy of training type with regard to resolving language barrier issues.

SPs are often used to portray unique cases; however, hiring SPs can be cost prohibitive for some clinical departments. Clinical doctors can serve as SPs to role-play with students because they have adequate personal experience in history-taking and can provide a sufficiently detailed medical history to create a meaningful learning experience for the students. However, because SPs were played by the faculty facilitators, there was potential for bias. In the future, professional SPs could be employed to resolve this problem. A lack of comparability between the histories taken in the sub-departments of obstetrics, gynaecology and reproductive endocrinology is also a limitation of the present study. Before intervention and after intervention groups will be included in future studies.

Another limitation of this study is that most students were from India; thus, we do not know if these results are generalizable to other student populations with different nationalities. Although there is more work to be done, our study indicates that students are willing to use such methods as adjuncts to current teaching strategies.

## Conclusions

Standardized patient training for practising history-taking can be included as part of the regular clinical training curriculum for foreign medical undergraduates in the Department of Obstetrics and Gynaecology in China.

## Additional files

Additional file 1: Questionnaire. (PDF 51 kb)

Additional file 2: Raw data. (XLSX 18 kb)

## Abbreviations

R: Real patient
SP: Standardized patient

## References

[1] The Joint commission. 2018 National Patient Safety Goals Presentation. https://www.jointcommission.org/standards_information/npsgs.aspx. Accessed 7 Dec 2017.

[2] Keifenheim KE, Teufel M, Ip J, Speiser N, Leehr EJ, Zipfel S (2015). Teaching history taking to medical students: a systematic review. BMC Med Educ.

[3] Brender E, Burke A, Glass RM (2005). JAMA patient page. Standardized patients. JAMA..

[4] Redelmeier DA, Schull MJ, Hux JE, Tu JV, Ferris LE (2001). Problems for clinical judgement: 1. Eliciting an insightful history of present illness. CMAJ..

[5] Barsky AJ (2002). Forgetting, fabricating, and telescoping: the instability of the medical history. Arch Intern Med.

[6] Bradley CP (2002). Review: interventions for health care providers improve provider-patient interactions and patient satisfaction. ACP J Club.

[7] Back AL, Arnold RM, Baile WF (2009). What makes education in communication transformative. J Cancer Educ.

[8] Cansever Z, Avsar Z, Tastan K (2015). Third year medical school students’ experiences of revealing patients’ stories through role playing. Eurasian J Med.

[9] Bokken L, Linssen T, Scherpbier A, van der Vleuten C, Rethans JJ (2009). Feedback by standardized patients in undergraduate medical education: a systematic review of the literature. Med Educ.

[10] Cleland JA, Abe K, Rethans JJ (2009). The use of standardized patients in medical education: AMEE guide no 42. Med Teach.

[11] von Lengerke T, Kursch A, Lange K, APG-Lehrteam MHH (2011). The communication skills course for second year medical students at Hannover Medical School: an evaluation study based on students' self-assessments. GMS Z Med Ausbild.

[12] Claramita M, Nugraheni MD, van Dalen J, van der Vleuten C (2013). Doctor-patient communication in Southeast Asia: a different culture?. Adv Health Sci Educ Theory Pract.

