# Supplementary material for: ‘Standardized patients’ in teaching the communication skill of history-taking to four-year foreign medical undergraduates in the department of obstetrics and gynaecology
Source: BMC Med Educ. 2019 Apr 15;19:108. doi: 10.1186/s12909-019-1541-y (PMC6466762; doi:10.1186/s12909-019-1541-y)
Supplement: Supplementary file 1 — Questionnaire. (PDF 51 kb) [file 12909_2019_1541_MOESM1_ESM.pdf]

Questionnaire

1. Which is the most difficult problem for you to take history from Chinese patients in OB/GY?
   A. Can’t take history by speaking Chinese Putong language
   B. Can’t understand the local language spoken by the Chinese patients
   C. Being unfamiliar with the OB/GY diseases
   D. Others such as____
2. Which method do you want to choose at first to practice taking history in OB/GY?
   A. Taking history from standard patients pretended by teachers
   B. Taking history from the real clinical patients
   C. Practicing Chinese language with teachers during the courses
   D. Watching some videos on taking history in OB/GY
3. Do you think the standard patients pretended by teachers can help you communicate with the Chinese patients?
   A. Yes, very useful　　B. Yes, partly　　C. No　　D. N/S
4. Which aspects do you think the standard patients can help you in?
   A. Training the skill in collecting history
   B. Practicing how to arrange the tests for the OB/GY patients
   C. Practicing making the diagnosis for the OB/GY patients
   D. Practicing treating the OB/GY patients
5. Which language do you want the teachers speak during the OB/GY clinical practice?
   A. English　　B. Both English and Chinese　　C. Chinese
6. Which country are you from? ______________________________
7. How long have you been in China? __________________Years
8. Which language do you usually speak in? ____________________
9. What suggestions can you supply to improve the teaching method of standard patients for taking history? ____
   ____
   ____
   ____
